# Systematic Review and Meta-analysis of the Association Between Radiation Therapy Treatment Volume and Patient Outcomes

**DOI:** 10.1016/j.ijrobp.2023.02.048

**Published:** 2023-12-01

**Authors:** Jerry Ye Aung Kyaw, Alice Rendall, Erin F. Gillespie, Tom Roques, Laurence Court, Yolande Lievens, Alison C. Tree, Chris Frampton, Ajay Aggarwal

**Affiliations:** ⁎ Guy's and St Thomas’ NHS Foundation Trust, London, United Kingdom; † Memorial Sloan Kettering Cancer Center, New York, New York; ‡ Norfolk and Norwich University Hospitals, Norwich, United Kingdom; § University of Texas MD Anderson Cancer Center, Houston, Texas, USA; ║ Department of Radiation Oncology, Ghent University Hospital and Ghent University, Ghent, Belgium; ¶ Royal Marsden NHS Foundation Trust and the Institute of Cancer Research, London, United Kingdom; # University of Otago, Christchurch, New Zealand; ⁎⁎ London School of Hygiene and Tropical Medicine, London, United Kingdom

## Abstract

**Purpose:**

Evidence of a volume–outcome association in cancer surgery has shaped the centralization of cancer services; however, it is unknown whether a similar association exists for radiation therapy. The objective of this study was to determine the association between radiation therapy treatment volume and patient outcomes.

**Methods and Materials:**

This systematic review and meta-analysis included studies that compared outcomes of patients who underwent definitive radiation therapy at high-volume radiation therapy facilities (HVRFs) versus low-volume facilities (LVRFs). The systematic review used Ovid MEDLINE and Embase. For the meta-analysis, a random effects model was used. Absolute effects and hazard ratios (HRs) were used to compare patient outcomes.

**Results:**

The search identified 20 studies assessing the association between radiation therapy volume and patient outcomes. Seven of the studies looked at head and neck cancers (HNCs). The remaining studies covered cervical (4), prostate (4), bladder (3), lung (2), anal (2), esophageal (1), brain (2), liver (1), and pancreatic cancer (1). The meta-analysis demonstrated that HVRFs were associated with a lower chance of death compared with LVRFs (pooled HR, 0.90; 95% CI, 0.87- 0.94). HNCs had the strongest evidence of a volume–outcome association for both nasopharyngeal cancer (pooled HR, 0.74; 95% CI, 0.62-0.89) and nonnasopharyngeal HNC subsites (pooled HR, 0.80; 95% CI, 0.75-0.84), followed by prostate cancer (pooled HR, 0.92; 95% CI, 0.86-0.98). The remaining cancer types showed weak evidence of an association. The results also demonstrate that some centers defined as HVRFs are undertaking very few procedures per annum (<5 radiation therapy cases per year).

**Conclusions:**

An association between radiation therapy treatment volume and patient outcomes exists for most cancer types. Centralization of radiation therapy services should be considered for cancer types with the strongest volume–outcome association, but the effect on equitable access to services needs to be explicitly considered.

## Introduction

The association between procedure volume and patient outcomes has been well established in cancer surgery for more than 4 decades.^1^ Several reviews have found that increased surgical volumes translate to improved survival.^2^, ^3^, ^4^, ^5^ This volume–outcome relationship has influenced how health systems are organized, with the selective centralization of cancer surgical services such as pancreatic, hepatobiliary, oesophageal and vascular surgery.^6^, ^7^, ^8^ However, evidence of a relationship between treatment volume and outcomes has yet to be convincingly demonstrated in the field of radiation therapy.

The global radiation therapy policy agenda has mostly focused on improving access to high-quality radiation therapy, given the evidence of significant disparities in utilization that directly affect cancer outcomes.^9^, ^10^, ^11^, ^12^, ^13^, ^14^, ^15^, ^16^, ^17^ More recently, it has been found that the use of more complex planning techniques such as stereotactic radiation therapy improves treatment outcomes, but owing to the infrastructure and expertise needed to integrate and implement innovative technologies and techniques, centralization is increasingly necessary to consolidate expertise.^18^, ^19^, ^20^

Radiation therapy services for rare tumor types such as sarcomas and pediatric tumors are already being centralized, as are techniques such as stereotactic radiosurgery for brain tumors.^21^ However, it remains unknown to what extent benefits can be accrued for different tumor types at a population level by centralization.

The importance of this question can also be seen in the increasing evidence of hospital-level variation in outcomes from radiation therapy. For instance, the UK National Prostate Cancer Audit has demonstrated a 2% to 20% variation in rates of moderate to severe gastrointestinal toxic effects after prostate radiation therapy across the 50 centers providing external beam radiation therapy in the English National Health Service.^22^ Given this level of variation, one aspect to consider is whether there is evidence of a volume–outcome relationship that may be driving better outcomes in higher-performing centers.

This systematic review and meta-analysis investigates whether there is evidence of an association between radiation therapy treatment volume and patient outcomes across different cancer types to support cancer service planning and quality improvement initiatives.

## Methods and Materials

### Search strategy

This systematic review was conducted according to Preferred Reporting Items for Systematic Reviews and Meta-Analyses (PRISMA) guidelines. The search was conducted for studies between January 1995 and February 2022, through MEDLINE and Embase databases via Ovid. Details of the search strategy are provided in Appendix E1.

### Study selection

**Fig. 1:**
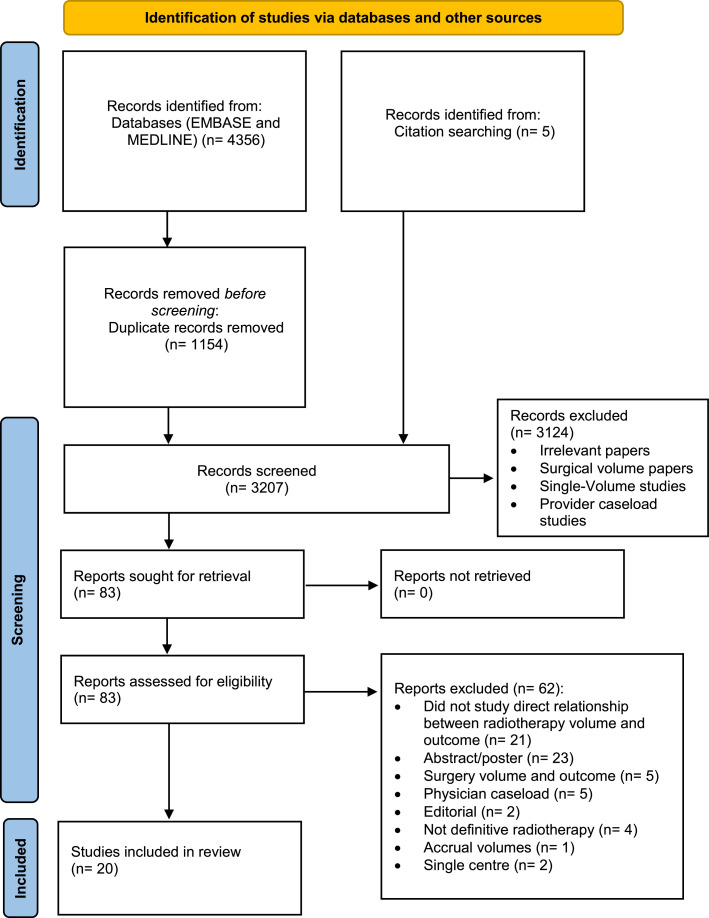
PRISMA (Preferred Reporting Items for Systematic Reviews and Meta-Analyses) diagram. Identification of studies was via Embase, MEDLINE, and citation searching for selected articles.

In addition to database searches through MEDLINE and Embase, references of identified articles were searched for further articles (Fig. 1). All articles included had to fulfill the following criteria: (1) all patients underwent definitive radiation therapy; (2) hospital volume was reported as a predictor variable; (3) a measurable endpoint such as overall survival, death, or complication or toxicity was clearly defined; (4) the study compared multiple high- and low-volume facilities (ie, ≥2 institutions each); and (5) articles were written in English. *Definitive radiation therapy* was defined as radiation therapy administered alone or with chemotherapy (chemoradiotherapy) with curative intent.

The review excluded studies that (1) compared single high-volume facilities with multiple low-volume facilities or vice versa; (2) defined volume at the level of individual oncologists, either as experience or workload, without any explicit mention of hospital volume; and/or (3) analyzed only patients who received adjuvant, neoadjuvant, or palliative radiation therapy.

### Data extraction

Data extracted included author(s), country, and year of the study; size of the study population; study period; study design; database analyzed; type and stage of cancer; radiation therapy modality or technique and dose; volume definition, including cutoffs; patient and hospital characteristics; statistical methods used; and primary and secondary outcome measures (Tables 1 and 2).

**Table 1:** Cancer types with 3 or more studies each

Study; year (country)	Data source (sample); period	Cancer type(s); stage	Primary RT modality/technique	Risk adjustment	Volume categorization/sensitivity analysis performed? (yes or no)	Endpoints
Nasopharyngeal cancer						
Ha et al; 2019^23^ (South Korea)	KROG (n = 1073); 1988-2011	Nasopharyngeal; AJCC stage: I-IV	3D-RT or IMRT 3D-CRT: mean dose of 67.99 Gy; IMRT: mean dose of 69.49 Gy	Age, sex, tumor category, lymph node category, clinical stage, WHO histologic classification, RT technique, ECOG performance status, MRI done, PET, concurrent chemotherapy	Categorical (dichotomized): Divided at a cutoff of an average of 10 cases per year over study period HV facilities: ≥10 cases per year LV facilities: <10 cases per year Propensity-matched: yes Sensitivity analysis performed: no	HV vs LV facilities • 5-y overall survival: 78.4% vs 62.7% (*P* < .001) • Adjusted overall survival rate over 120 mo: HR, 0.43; 95% CI, 0.32-0.57; *P* < .001 • 5-y LRPFS: 86.2% vs 65.8% (*P* < .001) • Adjusted LRPFS over 120 mo: HR, 0.34; 95% CI, 0.23-0.49; *P* < .001 • Acute grade ≥3 toxicities: hematologic toxicity (3.1% vs 19.2%, *P* < .001), mucositis (18.4% vs 22.0%, *P* = .003), xerostomia (0.9% vs 9.0%, *P* < .001)
Lai et al; 2020^24^ (Taiwan)	NHI (n = 16,315); 2001-2017	Nasopharyngeal; AJCC stage: I-IV	2D-RT or 3D-RT or IMRT Dose not specified	Age, sex, comorbidity, RT technique, hospital level, degree of urbanization, income, physician volume and age, physician sex, duration as certified radiation oncologist, geographic region of hospital, public hospital, accredited hospital level	Categorical (grouped): Divided into 4 quartiles based on cumulative volume of patients with NPC treated by a hospital over study period Q1: 1-85 cases Q2: 86-274 cases Q3: 275-651 cases Q4: ≥652 cases Propensity-matched: no Sensitivity analysis performed: no Volume effect seen on sensitivity analysis: no	• 5-y overall survival, Q1 vs Q2 vs Q3 vs Q4: 67.4% 73.3%, 76.0%, and 81.1%, respectively (*P* < .001) • Adjusted overall survival rate over 180 mo:Q1: 1.0 (reference)Q2: HR, 0.85; 95% CI, 0.78-0.93Q3: HR, 0.77; 95% CI, 0.68-0.88Q4: HR, 0.63; 95% CI, 0.54-0.74
Verma et al; 2018^3^ (USA)	NCDB (n = 4469); 2004-2013	Nasopharyngeal; AJCC stage: II-IV	CRT Dose ≥60 Gy	Age, race, clinical stage, tumor grade, Charlson-Deyo score, year of diagnosis, annual income, insurance type, location	Categorical (dichotomized): Divided at the 80th percentile based on total volume over study period HV facility: ≥11 cases over study period (top 20th) LV facility: <11 cases over study period (bottom 80th) Propensity-matched: yes Sensitivity analysis performed: yes Volume effect seen on sensitivity analysis: yes	HV vs LV facilities: • Difference in 5-y overall survival: 6% (95% CI, 3%-13%) • Adjusted overall survival rate over 120 mo: HR, 0.85; 95% CI, 0.75-0.96; *P* < .05
Yoshida et al; 2018^25^ (USA)	NCDB (n = 3941); 2004-2014	Nasopharyngeal; AJCC stage: II-IV	Conventional RT or IMRT or other RT All doses included with subgroup analysis on doses 65-81.6 Gy	Age, sex, race, tumor and lymph node stage, comorbidity score, academic center, insurance status, income, education, population of metropolitan area, chemotherapy, RT modality	Categorical (dichotomized): Divided at the 95th percentile based on annual facility volume HV facility: ≥3.36 cases per year (top 5th) LV facility: <3.36 cases per year (bottom 95th) Propensity-matched: yes Sensitivity analysis performed: yes Volume effect seen on sensitivity analysis: yes	HV vs LV facilities: • 5-y overall survival: 69.1% vs 63.3% (*P* = .003) (all doses); 72.1% vs 62.6% (*P* < .001) (doses 65-81.6 Gy; n = 2906) • Adjusted overall survival rate over 120 mo: HR, 0.79; 95% CI, 0.69-0.90; *P* < .001 (all doses); HR, 0.77; 95% CI, 0.65-0.90; *P* < .001 (doses 65-81.6 Gy)
Non-nasopharyngeal						
Chen et al; 2010^26^ (USA)	NCDB (n = 5690); 1996-2002	Laryngeal; AJCC stage: III-IV	CRT Dose not specified (technique not specified)	Age at diagnosis, sex, race, tumor stage, surgical or nonsurgical treatment, year of diagnosis, patient's residence, socioeconomic status, primary payer/insurance type at diagnosis, hospital type	Categorical (dichotomized): Divided at cutoffs based on median yearly facility volume for each facility type Teaching/research facilities HV facility: >7.1 cases per year LV facility: ≤7.1 cases per year Propensity-matched: no Sensitivity analysis performed: no	HV vs LV facilities (teaching/research facility): • 90-d mortality rate: 3% vs 6% • 365-d mortality rate: 17% vs 23% • 4-y mortality rate: 50% vs 63% • Adjusted overall survival over 90 mo: HR, 0.48; 95% CI, 0.31-0.75 • Adjusted overall survival over 365 d: HR, 0.72; 95% CI, 0.60-0.9 • Adjusted overall survival over 4 y: HR, 0.79; 95% CI, 0.69-0.90
David et al; 2017^27^ (USA)	NCDB (n = 46,567); 2004-2012	Larynx, oropharynx, hypopharynx; AJCC stage: III-IV	RT with/without CT All doses included, but subgroup analysis done on 65-81.6 Gy (technique not specified)	Age, sex, race, tumor and lymph node classification, anatomic site, comorbidity score, education level, income, insurance status, urban setting, concurrent chemotherapy	Categorical (dichotomized): Divided at the 99th percentile based on total volume over study period HV facility: top 1st LV facility: bottom 99th (cutoff values not given) Propensity-matched: yes Sensitivity analysis performed: yes Volume effect seen on sensitivity analysis: yes	HV vs LV facilities: • 5-y overall survival: 61.6% (95% CI, 59.8%-65.8%) vs 55.5% (95% CI, 53.6%-57.4%) (all doses) • Adjusted overall survival over 120 mo: HR, 0.798; 95% CI, 0.753-0.845; *P* < .001 (all doses); HR, 0.801; 95% CI, 0.747-0.858; *P* < .001 (doses 65-81.6 Gy; n = 35,195)
Both nasopharyngeal and nonnasopharyngeal head and neck cancers						
Tchelebi et al; 2021^28^ (USA)	NCDB (n = 16,540); 2004-2013	Larynx, tongue, tonsil, salivary gland, floor of mouth, hypopharynx, lip, oropharynx, and nasopharynx; clinical disease stage I-III and unknown	EBRT or BT No specific dose specified (palliative doses such as 30 Gy in 10 fractions, 20 Gy in 5 fractions, or 8 Gy in 1 fraction excluded)	Age, sex, race, clinical disease stage (0, 1, 2, 3, unknown), Charlson-Deyo comorbidity score, facility type, geographic area, annual household income, surgery performed, chemotherapy, immunotherapy, distance traveled to facility	Categorical (grouped): Q1: ≤1.5 cases per year (lowest volume) Q2: >1.5-3 cases per year Q3: >3-5.6 cases per year Q4: ≥5.6 cases per year (highest volume) Propensity-matched: no Sensitivity analysis performed: no	Adjusted 5-y overall survival per volume quartile (reference Q1 = 1.0): • Q2 vs Q1: HR, 0.86 (95% CI, 0.82-0.96; *P* < .001) • Q3 vs Q1: HR, 0.83 (95% CI, 0.76-0.90; *P* < .001) • Q4 vs Q1: HR, 0.82 (95% CI, 0.75-0.90; *P* < .001)
Cervical cancer						
Lin et al; 2014^29^ (USA)	NCDB (n = 27,660); 1998-2010	Cervical cancer; FIGO/AJCC stage: IIB-IIIB	EBRT with/without CT and/or BT Dose not specified (EBRT: technique not specified)	Age, race, Hispanic ethnicity, median income, urban setting, insurance status, Charlson-Deyo score, FIGO/AJCC clinical stage, histology, tumor size	Categorical (grouped): Divided into 4 quartiles based on total number of cases treated at each facility over the reporting period Q1: ≤2.3 cases per year (lowest volume) Q2: 2.4-5.1 cases per year Q3: 5.2-9.3 cases per year Q4: ≥9.4 cases per year (highest volume) Propensity-matched: no Sensitivity analysis performed: no	Median overall survival (mo) (*P* < .0005): • Q1: 37.8 (95% CI, 35.2-40.3) • Q2: 45.0 (95% CI, 41.8-48.3) • Q3: 49.1 (95% CI, 45.9-52.2) • Q4: 51.5 (95% CI, 47.7-55.4) Adjusted overall survival per volume quartile over 100 mo (reference Q1 = 1.0): • Q2 vs Q1: HR, 0.96 • Q3 vs Q1: HR, 0.92 • Q4 vs Q1: HR, 0.88 HR, 0.96/quartile increase (95% CI, 0.93-0.99; *P* < .0005)
Lin et al; 2018^30^ (Taiwan)	TCR (n = 2582); 2007-2013	Cervical cancer; FIGO stage: IB-IVA	EBRT with/without CT and/or BT Dose: ≥34 Gy or ≥60 Gy with brachytherapy boost (EBRT: technique not specified)	Age, tumor histology, FIGO stage, lymph node status, tumor size, intracavity brachytherapy boost status, concurrent chemotherapy	Categorical (grouped): Divided into 3 categories based on annual case load per facility T1: ≤2 cases per year (lowest volume) T2: 3-5 cases per year T3: ≥5 cases per year (highest volume) Propensity-matched: no Sensitivity analysis performed: no	• 5-y overall survival (T1 vs T2 vs T3): 37% vs 46% vs 63%; *P* < .001 • Adjusted overall survival rate per tertile over 96 mo (reference T1 = 1.0): T2 vs T1: HR, 1.02; 95% CI, 0.74-1.40; *P* = .90; T3 vs T1: HR, 0.82; 95% CI, 0.61-1.11; *P* = .21 (significant interaction by tumor size and FIGO staging noted) • Adjusted overall survival rate over 96 mo stratified by tumor size and FIGO stage: i. Tumor ≤4 cm T3 vs (T1 + T2): HR, 0.94; 95% CI, 0.77-1.16; *P* = .59 ii. Tumor >4 cm T3 vs (T1 + T2): HR, 0.74; 95% CI, 0.62-0.88; *P* < .01 ii. FIGO stage IB to IIA T3 vs (T1 + T2): HR, 0.82; 95% CI, 0.61-1.10; *P* = .19 iv. FIGO stage IIB to IVA T3 vs (T1 + T2): HR, 0.78; 95% CI, 0.67-0.90; *P* < .01
Tchelebi et al; 2021^28^ (USA)	NCDB (n = 2788); 2004-2013	Cervical cancer; clinical disease stage I-III and unknown	EBRT or BT No specific dose noted (palliative doses such as 30 Gy in 10 fractions, 20 Gy in 5 fractions, or 8 Gy in 1 fraction excluded)	Age, sex, race, clinical disease stage (0, 1, 2, 3, unknown), Charlson-Deyo comorbidity score, facility type, geographic area, annual household income, surgery performed, chemotherapy, immunotherapy, distance traveled to facility	Categorical (grouped): Q1: <0.4 cases per year (lowest volume) Q2: 0.4 to <0.9 cases per year Q3: 0.9 to <1.5 cases per year Q4: ≥1.5 cases per year (highest volume) Propensity-matched: no Sensitivity analysis performed: no	Adjusted 5-y overall survival per volume quartile (reference Q1 = 1.0): • Q2 vs Q1: HR, 0.95 (95% CI, 0.79-1.15; *P* < .62) • Q3 vs Q1: HR, 0.92 (95% CI, 0.74-1.14; *P* < .43) • Q4 vs Q1: HR, 0.85 (95% CI, 0.68-1.07; *P* < .17)
Wright et al; 2015^31^ (USA)	NCDB (n = 12,048); 1998-2011	Cervical cancer; FIGO stage: IIB-IVA	EBRT with/without CT and/or BT Dose not specified (EBRT: technique not specified)	Age, race, insurance, clinical tumor grade, stage, histology, hospital region, location, type of hospital	Continuous (annualized) Categorical (grouped) Total number of patients treated and divided by the number of years in which a hospital treated at least 1 patient with locally advanced cervical cancer Divided into 4 quartiles based on annualized case volumes: Q1: <2 cases per year (lowest volume) Q2: 2-3.99 cases per year Q3: 4-5.99 cases per year Q4: ≥6 cases per year (highest volume) Propensity-matched: no Sensitivity analysis performed: yes Volume effect seen on sensitivity analysis: no	1. Adjusted overall survival (continuous) variable • 5-y overall survival: HR, 0.99 (95% CI, 0.98-1.00; *P* < .05) 2. Overall survival per volume quartile over 120 mo (reference Q1 = 1.0) (*P* > .05) • Q2 vs Q1: HR, 1.01 (95% CI, 0.92-1.11) • Q3 vs Q1: HR, 0.97 (95% CI, 0.90-1.05) • Q4 vs Q1: HR, 0.91 (95% CI, 0.82-1.02)
Prostate cancer						
Chen et al; 2016^4^ (USA)	NCDB (n = 19,656); 2004- 2006	Prostate cancer; TNM stage: T1-4, N (unspecified), M0; Gleason score: 1-10	EBRT and/or BT Dose not specified (technique not specified)	Age, race, Gleason score, Charlson-Deyo comorbidity score, tumor stage, PSA status, radiation type, ADT, hospital setting, insurance status, household income, residence type, education level, hospital setting, RT technique	Continuous (cumulative) Categorical (dichotomized) 1. Volume as a continuous variable based on the cumulative radiation-treated prostate cancer at each facility from 2004 to 2006 (every 100 patients increment) 2. Divided at the 80th percentile based on annual facility volume HV facilities: ≥43 cases per year (top 20th) LV facilities: <43 cases per year (bottom 80th) Propensity-matched: yes Sensitivity analysis performed: yes Volume effect seen on sensitivity analysis: yes	1. Adjusted overall survival over 108 mo (continuous variable) • Increasing by 100 radiation-managed patients: HR, 0.97; 95% CI, 0.95-0.98; *P* < .0001 2. HV vs LV facilities (categorical variable) • 7-y overall survival: 76% vs 74%, *P* < .0005 • Adjusted overall survival over 108 mo: HR, 0.91; 95% CI, 0.86-0.96, *P* < .0005
Patel et al; 2020^32^ (USA)	NCDB (n = 1899); 2004-2016	Prostate cancer; TNM stage: T1-4, N1, M0; Gleason score: 6-10	EBRT and ADT Dose ≥60 Gy (technique not specified)	Age, race, tumor stage, Gleason score, PSA level, Charlson-Deyo score, percentage residence without high school degree, median income quartiles, total radiation dose, boost radiation dose, year of diagnosis, distance to facility	Categorical (dichotomized): Divided at a cutoff of 67 average cumulative cases per facility per year from 2004 to the time of diagnosis for a patient HV facilities: ≥67 cases per year LV facilities: <67 cases per year Propensity-matched: yes Sensitivity analysis performed: no	HV vs LV facilities: • Median overall survival: 111.1 mo (95% CI, 101.5-127.9) vs 94.5 mo (95% CI, 88.2-105.8)( *P* = .04) • 10-y overall survival: 44.7% (95% CI, 37.7%-51.6%) vs 35.6% (95% CI, 30.1%-41.1%) • Adjusted overall survival over 168 mo: HR, 0.80; 95% CI, 0.67-0.99; *P* = .04
Tchelebi et al; 2021^28^ (USA)	NCDB (n = 38,296); 2004-2013	Prostate cancer; clinical disease stage I-III and unknown	EBRT or BT No specific dose noted (palliative doses such as 30 Gy in 10 fractions, 20 Gy in 5 fractions, or 8 Gy in 1 fraction excluded)	Age, sex, race, clinical disease stage (0, 1, 2, 3, unknown), Charlson-Deyo comorbidity score, facility type, geographic area, annual household income, surgery performed, chemotherapy, immunotherapy, distance traveled to facility	Categorical (grouped): Q1: ≤3.9 cases per year (lowest volume) Q2: 3.9 to <7.2 cases per year Q3: 7.2 to <13 cases per year Q4: ≥13 cases per year (highest volume) Propensity-matched: no Sensitivity analysis performed: no	Adjusted 5-y overall survival per volume quartile (reference Q1 = 1.0): • Q2 vs Q1: HR, 0.97 (95% CI, 0.87-1.07; *P* < .51) • Q3 vs Q1: HR, 0.91 (95% CI, 0.82-1.01; *P* < .08) • Q4 vs Q1: HR, 0.82 (95% CI, 0.74-0.91; *P* < .001)
Chen et al; 2009^33^ (USA)	SEER (n = 5595); 1991-1999	Prostate cancer; TNM stage: T1-3, N0-N1, M0; Gleason score: 2 and greater	BT dose not specified	Age, race, urban residence, marital status, income status, cancer grade, tumor grade, lymph node status, PSA status, Gleason score, brachytherapy year, concomitant EBRT, ADT, history of TURP and IBD	Continuous (cumulative): Volume as a continuous variable based on the total number of brachytherapy procedures performed from 1991 to 2001 Analyzed volume for every increase in 100 brachytherapy procedures performed at a facility Propensity-matched: no Sensitivity analysis performed: no	HV vs LV facilities: 1. Complication rates over 10-y study period • Rate of invasive complication procedures within 2 y of RT: OR, 1.01/100 additional cases (95% CI, 0.94-1.09; *P* = .70) • Rate of combined complication diagnosis and invasive procedures:OR, 0.94/100 additional cases (95% CI, 0.91-0.98; *P* < .001) 2. Cancer recurrence over 10-y study period: HR, 0.99; 95% CI, 0.96-1.02; *P* = .66 3. Prostate cancer death over 10-y study period: HR, 1.07; 95% CI, 0.98-1.17; *P* = .14 4. All deaths over 10-y study period: HR, 0.99; 95% CI, 0.96-1.02; *P* = .48
Bladder cancer						
Bajaj et al; 2017^34^ (USA)	NCDB (n = 2763); 2004-2013	Muscle-invasive bladder cancer; TNM stage: T2-4, N0-3, M0	RT with/without CT (technique not specified) Dose: 60-70 Gy	Age, sex, race, facility type, median case volume, ethnicity, Charlson-Deyo comorbidity score, insurance, median income, education level, census region, metropolitan area, year group, tumor grade, chemotherapy type, tumor group, radiation therapy dose, distance to hospital, extent of resection	Categorical (dichotomized): Divided at the 75th percentile based on median case volume per facility over study period (10 y) HV facilities: top 25th LV facilities: bottom 75th Propensity-matched: no Sensitivity analysis performed: yes Volume effect seen on sensitivity analysis: no	HV vs LV facilities: • Adjusted overall survival over 100 mo: HR, 0.99; 95% CI, 0.94-1.04; *P* = .60
Fischer-Valuck et al; 2019^35^ (USA)	NCDB (n = 1635); 2004-2013	Muscle-invasive bladder cancer; TNM stage: T2-4, N0, M0	CRT Dose: 50.4-75 Gy	Age, sex, race, tumor stage, Charlson-Deyo comorbidity, treatment facility type, radiation dose, radiation fractionation, number of chemotherapy agents, year of diagnosis, treatment facility location, insurance status, population setting, household income, education level, (all patients underwent TURBT before RT)	Categorical (dichotomized): Divided at the 70th percentile based on the number of bladder preservation cases completed at each facility over the study period (10 y) HV facilities: ≥6 cases (top 30th) LV facilities: <6 cases (bottom 70th) Propensity-matched: yes Sensitivity analysis performed: yes Volume effect seen on sensitivity analysis: yes	HV vs LV facilities: • Median length of survival (mo): 36.1 (95% CI, 26.5-45.8) vs 28.1 (95% CI, 23.9-32.3) (all doses); 39.1 (95% CI, 29.3-48.8) vs 30.7 (95% CI, 27.6-33.7) (doses 59.4-64.8 Gy; n = 1213) • Adjusted overall survival over 120 mo: HR, 0.82; 95% CI, 0.70-0.96; *P* = .016 (all doses); HR, 0.83; 95% CI, 0.73-0.96; *P* = .037 (doses 59.4-64.8 Gy; n = 1213)
D'Rummo et al; 2019^36^ (USA)	NCDB (n = 7562); 2004-2015	Muscle-invasive bladder cancer; TNM stage: T2-4, 0-3, M0	EBRT with/without CT (technique not specified) RT dose >30 Gy	Age, sex, race, primary insurance, median household income, education level, residence type, Charlson-Deyo comorbidity score, tumor stage, lymph node status, hospital setting	Categorical (dichotomized): Divided at the 80th percentile based on cumulative number of cases for all facilities during the study period (12 y) HV facilities: ≥14 cases (top 20th) LV facilities: <14 cases (bottom 80th) Propensity-matched: no Sensitivity analysis performed: yes Volume effect seen on sensitivity analysis: yes	HV vs LV facilities: • 5-y overall survival: 24.8% vs 20.7%; *P* = .013 (all doses) • Overall survival remained significantly greater in HV facilities (*P* = .0081) (doses 55-66 Gy); no value given in study

*Abbreviations*: 2D = 2-dimensional; 3D = 3-dimensional; 3D-CRT = 3-dimensional conformal radiation therapy; ADT = androgen deprivation therapy; AJCC = American Joint Committee on Cancer; BT = brachytherapy; CI = confidence interval; CRT = chemoradiotherapy; CT = chemotherapy; EBRT = external beam radiation therapy; ECOG = Eastern Cooperative Oncology Group; FIGO = International Federation of Gynecology and Obstetrics; HR = hazard ratio; HV = high volume; IBD = inflammatory bowel disease; IMRT = intensity modulated radiation therapy; KROG = Korean Radiation Oncology Group; LRPFS = locoregional progression-free survival; LV = low volume; MRI = magnetic resonance imaging; NCDB = National Cancer Database; NHI = National Health Insurance; NPC = nasopharyngeal cancer; OR = odds ratio; PET = positron emission tomography; PSA = prostate-specific antigen; RT = radiation therapy; SEER = Surveillance, Epidemiology, and End Results; TCR = Taiwan Cancer Registry; TURBT = transurethral resection of bladder tumor; TURP = transurethral resection of the prostate; WHO = World Health Organization.

**Table 2:** Other types of cancers with fewer than 3 studies each

Study; year (country)	Data source (sample); period	Cancer type(s); stage	Primary RT modality/technique	Risk adjustment	Volume categorization/ sensitivity analysis performed? (yes or no)	Endpoints
Lung cancer						
Wang et al; 2015^37^ (USA)	NCDB (n = 10,072); 2004-2006	Non-small cell lung cancer; AJCC: stage III	3D-RT or IMRT or nonconformal RT dose: 59.4-74 Gy	Age, race, median income, insurance status, geographic region, patient location, travel distance to reporting facility, Charlson-Deyo comorbidity score, RT modality, total RT dose fractionation, year of diagnosis	Categorical (dichotomized): Divided at the 90th percentile based on the average annual case volume per facility HV facilities: ≥12 cases per year (top 10th) LV facilities: <12 cases per year (bottom 90th) Propensity-matched: yes Sensitivity analysis performed: yes Volume effect seen on sensitivity analysis: no	HV vs LV facilities: • Overall median survival times (mo): 19.7 (95% CI, 18.3-20.9) vs 17.3 (95% CI, 16.9-17.8) • Adjusted overall survival rate over 60 mo: HR, 0.91; 95% CI, 0.84-0.99; *P* = .04
Tchelebi et al; 2021^28^ (USA)	NCDB (n = 28,180); 2004-2013	Non-small cell lung cancer; clinical disease stage I-III and unknown	EBRT or BT No specific dose noted (palliative doses such as 30 Gy in 10 fractions, 20 Gy in 5 fractions, or 8 Gy in 1 fraction excluded)	Age, sex, race, clinical disease stage (0, 1, 2, 3, unknown), Charlson-Deyo comorbidity score, facility type, geographic area, annual household income, surgery performed, chemotherapy, immunotherapy, distance traveled to facility	Categorical (grouped): Q1: <2.7 cases per year (lowest volume) Q2: 2.7 to <5.5 cases per year Q3: 5.5 to <8.5 cases per year Q4: ≥8.5 cases per year (highest volume) Propensity-matched: no Sensitivity analysis performed: no	Adjusted 5-y overall survival per volume quartile (reference Q1= 1.0): • Q2 vs Q1: HR, 0.98 (95% CI, 0.93-1.02; *P* < .31) • Q3 vs Q1: HR, 0.95 (95% CI, 0.9-0.99; *P* < .02) • Q4 vs Q1: HR, 0.89 (95% CI, 0.84-0.93; *P* < .001)
Tchelebi et al; 2021^28^ (USA)	NCDB (n = 4325); 2004-2013	Small cell lung cancer; clinical disease stage I-III and unknown	EBRT or BT No specific dose noted (palliative doses such as 30 Gy in 10 fractions, 20 Gy in 5 fractions, or 8 Gy in 1 fraction excluded)	Age, sex, race, clinical disease stage (0, 1, 2, 3, unknown), Charlson-Deyo comorbidity score, facility type, geographic area, annual household income, surgery performed, chemotherapy, immunotherapy, distance traveled to facility	Categorical (grouped): Q1: <0.5 cases per year (lowest volume) Q2: 0.5 to <0.9 cases per year Q3: 0.9 to <1.4 cases per year Q4: ≥1.4 cases per year (highest volume) Propensity-matched: no Sensitivity analysis performed: no	Adjusted 5-y overall survival per volume quartile (reference Q1= 1.0): • Q2 vs Q1: HR, 0.96 (95% CI, 0.86-1.07; *P* < .47) • Q3 vs Q1: HR, 0.98 (95% CI, 0.87-1.1; *P* < .73) • Q4 vs Q1: HR, 1.01 (95% CI, 0.9-1.13; *P* < .93)
Cancer of the anus						
Amini et al; 2017^38^ (USA)	NCDB (n = 13,016); 2004-2013	Anal squamous cell carcinoma; AJCC stage: I-III	IMRT or 3D-RT with/without CT Dose not specified	Age, AJCC clinical staging, use of chemotherapy, facility type, distance to radiation facility, insurance status, residence, Charlson-Deyo comorbidity score, year of diagnosis, duration of RT	Categorical (grouped): Divided into tertiles T1: Low-volume facility T2: Intermediate-volume facility T3: High-volume facility Propensity-matched: yes Sensitivity analysis performed: no	1. All RT modalities5-y overall survival: • T1 vs T2 vs T3: 70.1% vs 71.4% vs 74.6% (*P* < .001) Adjusted overall survival rate per tertile over 120 mo: • T2: HR, 0.94; 95% CI, 0.86-1.05; *P* = .302 • T3: HR, 0.79; 95% CI, 0.69-0.91; *P* = .001 2. Only IMRT (n = 4551) Adjusted overall survival rate over 120 mo (reference T1 = 1.0): • T2: HR, 0.81; 95% CI, 0.67-0.99; *P* = .035 • T3: HR, 0.76; 95% CI, 0.62-0.94; *P* = .009
Tchelebi et al; 2021^28^ (USA)	NCDB (n = 2236); 2004-2013	Cancer of the anus (type not specified); clinical disease stage I-III and unknown	EBRT or BT No specific dose noted (palliative doses such as 30 Gy in 10 fractions, 20 Gy in 5 fractions, or 8 Gy in 1 fraction excluded)	Age, sex, race, clinical disease stage (0, 1, 2, 3, unknown), Charlson-Deyo comorbidity score, facility type, geographic area, annual household income, surgery performed, chemotherapy, immunotherapy, distance traveled to facility	Categorical (grouped): Q1: <0.3 cases per year (lowest volume) Q2: 0.3 to <0.5 cases per year Q3: 0.5 to <0.8 cases per year Q4: ≥0.8 cases per year (highest volume) Propensity-matched: no Sensitivity analysis performed: no	Adjusted 5-y overall survival per volume quartile (reference Q1= 1.0): • Q2 vs Q1: HR, 0.83 (95% CI, 0.64-1.08; *P* < .17) • Q3 vs Q1: HR, 1.07 (95% CI, 0.83-1.37; *P* < .6) • Q4 vs Q1: HR, 0.98 (95% CI, 0.76-1.27; *P* < .91)
Brain cancer						
Haque et al; 2017^39^ (USA)	NCDB (n = 4892); 2006-2012	Glioblastoma; grading not mentioned	EBRT and CT Dose: 59.4-60 Gy (EBRT: technique not specified)	Age, race, sex, Charlson-Deyo comorbidity score, year of diagnosis, income, insurance, surgery type, county, location	Categorical (grouped): Divided into 4 quartiles according to mean annual volume for each facility over the study period Q1: ≤3.9 cases per year (lowest volume) Q2: 4.0-6.1 cases per year Q3: 6.3-8.7 cases per year Q4: ≥9.1 cases per year (highest volume) Propensity-matched: no Sensitivity analysis performed: no	Median survival (mo): • HV (Q4) vs LV (Q1) facilities: 16.5 vs 14.1 mo (*P* < .001) Adjusted overall survival rate per quartile over 100 mo (reference Q4 = 1.0): • Q1: HR, 1.096; 95% CI, 1.005-1.197; *P* = .039 • Q2: HR, 1.089; 95% CI, 0.996-1.191; *P* = .061 • Q3: HR, 1.047; 95% CI, 0.958-1.144; *P* = .312
Tchelebi et al; 2021^28^ (USA)	NCDB (n = 2062); 2004-2013	Brain cancer (type not specified); clinical disease stage I-III and unknown	EBRT or BT No specific dose noted (palliative doses such as 30 Gy in 10 fractions, 20 Gy in 5 fractions, or 8 Gy in 1 fraction excluded)	Age, sex, race, clinical disease stage (0, 1, 2, 3, unknown), Charlson-Deyo comorbidity score, facility type, geographic area, annual household income, surgery performed, chemotherapy, immunotherapy, distance traveled to facility	Categorical (grouped): Q1: ≤0.3 cases per year (lowest volume) Q2: >0.3 to 0.7 cases per year Q3: >0.7 to 1.2 cases per year Q4: >1.2 cases per year (highest volume) Propensity-matched: no Sensitivity analysis performed: no	Adjusted 5-y overall survival per volume quartile (reference Q1= 1.0): • Q2 vs Q1: HR, 0.93 (95% CI, 0.80-1.09; *P* < .39) • Q3 vs Q1: HR, 0.96 (95% CI, 0.80-1.14; *P* < .6) • Q4 vs Q1: HR, 0.98 (95% CI, 0.82-1.17; *P* < .82)
Esophageal cancer						
Tchelebi et al; 2021^28^ (USA)	NCDB (n = 26,709); 2004-2013	Esophageal cancer; clinical disease stage I-III and unknown	EBRT or BT No specific dose noted (palliative doses such as 30 Gy in 10 fractions, 20 Gy in 5 fractions, or 8 Gy in 1 fraction excluded)	Age, sex, race, clinical disease stage (0, 1, 2, 3, unknown), comorbidity, facility type, insurance, geographical area, annual household income, surgery, chemotherapy, immunotherapy, distance to facility	Categorical (grouped): Q1: <0.3 cases per year (lowest volume) Q2: 0.3 to <0.5 cases per year Q3: 0.5 to <1 cases per year Q4: ≥1 cases per year (highest volume) Propensity-matched: no Sensitivity analysis performed: no	Adjusted 5-y overall survival per volume quartile (reference Q1 = 1.0): • Q2 vs Q1: HR, 0.89 (95% CI, 0.77-1.04; *P* < .14) • Q3 vs Q1: HR, 0.94 (95% CI, 0.81-1.09; *P* < .39) • Q4 vs Q1: HR, 0.88 (95% CI, 0.75-1.03; *P* < .12)
Liver cancer						
Holliday et al; 2017^40^ (USA)	NCDB (n = 3579); 2004-2014	Hepatocellular carcinoma (node-negative); AJCC stage: I-III	EBRT or BT Dose not specified (EBRT: technique not specified)	Age, race, sex, clinical stage, tumor stage, Charlson-Deyo comorbidity index, bilirubin level, creatinine level, INR level, alpha-foetoprotein levels at diagnosis, tumor size, median income, insurance, facility type	Categorical (dichotomized): Divided at the 90^th^ percentile based on average annual number of HCC over study period HV facility: >144 cases per year (top 10th) LV facility: ≤144 cases per year (bottom 90th) Propensity-matched: no Sensitivity analysis performed: no	HV vs LV facilities: • Adjusted overall survival rate over 120 mo: HR, 1.349; 95% CI, 0.967-1.881; *P* = .078
Pancreatic cancer						
Tchelebi et al; 2021^28^ (USA)	NCDB (n = 2075); 2004-2013	Pancreatic cancer; clinical disease stage I-III and unknown	EBRT or BT No specific dose noted (palliative doses such as 30 Gy in 10 fractions, 20 Gy in 5 fractions, or 8 Gy in 1 fraction excluded)	Age, sex, race, clinical disease stage (0, 1, 2, 3, unknown), Charlson-Deyo comorbidity score, facility type, geographic area, annual household income, surgery performed, chemotherapy, immunotherapy, distance traveled to facility	Categorical (grouped): Q1: ≤0.3 cases per year (lowest volume) Q2: >0.3 to 0.7 cases per year Q3: >0.7 to 1.4 cases per year Q4: >1.4 cases per year (highest volume) Propensity-matched: no Sensitivity analysis performed: no	Adjusted 5-y overall survival per volume quartile (reference Q1= 1.0): • Q2 vs Q1: HR, 1.05 (95% CI, 0.90-1.22; *P* < .56) • Q3 vs Q1: HR, 0.87 (95% CI, 0.75-1.02; *P* < .09) • Q4 vs Q1: HR, 0.84 (95% CI, 0.71-0.98; *P* < .01)

*Abbreviations*: 2D = 2-dimensional; 3D = 3-dimensional; 3D-CRT = 3-dimensional conformal radiation therapy; AJCC = American Joint Committee on Cancer; BT = brachytherapy; CI = confidence interval; CRT = chemoradiotherapy; CT = chemotherapy; EBRT = external beam radiation therapy; HCC = hepatocellular cancer; HR = hazard ratio; HV = high volume; IMRT = intensity modulated radiation therapy; INR = international normalized ratio; IV = intermediate volume; LV = low volume; NCDB = National Cancer Database; RT = radiation therapy.

### Quality of studies

The quality of studies was reviewed using the Newcastle-Ottawa Scale, with discrepancies reviewed after joint article review and discussion. A study with a score of 7 to 9 was considered low risk of bias; 4 to 6, moderate risk of bias; and 0 to 3, high risk of bias. Importantly, a few key predictors needed to be adjusted for in all studies, such as age, sex (where applicable), comorbidity, clinical staging of disease, radiation therapy technique, insurance, and socioeconomic variables such as income and education. The use of propensity-scored matching and sensitivity analysis was also taken into consideration when evaluating results (Table 3).

**Table 3:** Newcastle-Ottawa quality assessment scores

Study; year	Representativeness of the exposed cohort	Selection of nonexposed cohort	Ascertainment of exposure	Demonstration that outcome of interest was not present at start of study	Comparability of cohorts (maximum 2 stars)	Assessment of outcome	Was follow-up long enough for outcomes to occur?	Adequacy of follow-up cohorts	Total score
Nasopharyngeal cancer									
Verma et al; 2018^3^	**★**	**★**	**★**	**★**	**★**	**★**	**★**		7/9
Yoshida et al; 2018^25^	**★**	**★**	**★**	**★**	**★★**	**★**	**★**		8/9
Ha et al; 2019^23^		**★**	**★**	**★**	**★**	**★**	**★**		6/9
Lai et al; 2020^24^	**★**	**★**	**★**	**★**	**★**	**★**	**★**		7/9
Laryngeal, oropharyngeal, hypopharyngeal, and oral cavity cancer									
Chen et al; 2010^26^	**★**	**★**	**★**	**★**	**★**	**★**	**★**	**★**	8/9
David et al; 2017^27^	**★**	**★**	**★**	**★**	**★**	**★**	**★**		7/9
Both nasopharyngeal and nonnasopharyngeal head and neck cancers									
Tchelebi et al; 2021^28^	**★**	**★**	**★**	**★**	**★**	**★**	**★**		7/9
Cervical cancer									
Lin et al; 2014^29^	**★**	**★**	**★**	**★**	**★**	**★**	**★**		7/9
Wright et al;2015^31^	**★**	**★**	**★**	**★**	**★**	**★**	**★**	**★**	8/9
Lin et al; 2018^30^	**★**	**★**	**★**	**★**	**★**	**★**	**★**		7/9
Tchelebi et al; 2021^28^	**★**	**★**	**★**	**★**	**★**	**★**	**★**		7/9
Prostate cancer									
Chen et al; 2009^33^	**★**	**★**	**★**	**★**	**★★**	**★**	**★**		8/9
Chen et al; 2016^4^	**★**	**★**	**★**	**★**	**★★**	**★**	**★**		8/9
Patel et al; 2020^32^	**★**	**★**	**★**	**★**	**★★**	**★**	**★**		8/9
Tchelebi et al; 2021^28^	**★**	**★**	**★**	**★**	**★**	**★**	**★**		7/9
Bladder cancer									
Bajaj et al; 2017^28^	**★**	**★**	**★**	**★**	**★**	**★**	**★**		7/9
Fischer-Valuck et al; 2019^35^	**★**	**★**	**★**	**★**	**★**	**★**	**★**		7/9
D'Rummo et al; 2019^36^	**★**	**★**	**★**	**★**	**★**	**★**	**★**		7/9
Lung, liver, brain, anal, esophageal, and pancreatic cancer									
Wang et al; 2015^37^	**★**	**★**	**★**	**★**	**★★**	**★**	**★**		8/9
Holliday et al; 2017^40^	**★**	**★**	**★**	**★**	**★**	**★**	**★**		7/9
Haque et al; 2017^39^	**★**	**★**	**★**	**★**	**★**	**★**	**★**		7/9
Amini et al; 2017^38^	**★**	**★**	**★**	**★**	**★**	**★**	**★**		7/9
Tchelebi et al; 2021^28^	**★**	**★**	**★**	**★**	**★**	**★**	**★**		7/9

### Meta-analysis

Studies either dichotomized volume groups or split volume groups into tertiles or quartiles. Our meta-analysis maintained the volume categories used in the studies and was based on the hazard ratios (HRs) provided in the studies. The volume definitions for each of the studies are summarized in Tables 1 and 2 and can also be found in the results.

For the studies that categorized volume into tertiles and quartiles, the meta-analysis only used the HR of the lowest-volume group (reference) and the next lowest group to determine the most conservative pooled radiation therapy volume–outcome relationship. This was because a comparison of the groups with highest and lowest volume may overstate the size of the association, especially given the potential for residual confounding in routine observational data. If a significant volume–outcome association between the lowest-volume group and the next lowest group was evident, we would reasonably expect there to be a volume association when comparing the lowest-volume group with higher-volume groups.

The HR estimates for each cancer type from each study were entered into RevMan, version 5.4, for meta-analysis. A random-effects model was used for the summaries, and pooled estimates were generated for each cancer type and overall types. The results of the meta-analysis assessing the association between radiation therapy procedure volume and outcome were categorized by tumor types. Specification of volume thresholds and outcomes are described under each cancer type in this review (Table 4 shows radiation therapy modalities/techniques).

**Table 4:** Details of radiation therapy characteristics

Cancer groups	Radiation therapy characteristics
Head and neck cancers	Only 4 studies^23^^,^^25^^,^^24^^,^^28^ (n = 4/7) described radiation therapy techniques used. Yoshida et al^25^ categorized patients into those who received conventional radiation therapy: 2-dimensional radiation therapy (2D-RT), intensity modulated radiation therapy (IMRT), and other forms of radiation therapy (techniques not specified). They also analyzed volume–outcome relationship for all doses as well as a subgroup of patients who received doses of 65-81.6 Gy.^25^ Ha et al^23^ grouped patients into those who received 3-dimensional conformal radiation therapy (3D-CRT) at mean doses of 67.99 Gy or IMRT at mean doses of 69.49 Gy. Lai et al^24^ grouped patients into those receiving either 2D-RT, 3D-CRT, or IMRT^25^ but did not mention doses. Tchelebi et al^28^ included only patients who received definitive radiation therapy doses via external beam radiation therapy (EBRT; technique not specified) and/or brachytherapy; however, doses were not mentioned. Among those who did not specify radiation therapy technique, Verma et al^3^ included only radiation therapy doses >60 Gy and David et al^27^ included all doses of radiation therapy and conducted a subgroup analysis on those who received 65-81.6 Gy. Chen et al^26^ did not specify radiation therapy doses (Table 1).
Cervical cancer	All 4 studies included patients receiving a combination of EBRT (technique used not specified) with/without chemotherapy and/or brachytherapy. Only Lin et al^30^ stated that patients treated primarily with curative radiation therapy were included (≥34 Gy or ≥60 Gy with brachytherapy boost). Lin et al^29^ and Wright et al^31^ did not give dose information and did not state whether definitive radiation therapy was given to patients, which may mean that those 2 studies included patients exposed to a wider range of doses (Table 1). Tchelebi et al^28^ included patients who received definitive radiation therapy doses; however, specific doses were not mentioned.
Prostate cancer	All studies did not mention what technique of EBRT was used, for example, IMRT or 3D-CRT. Chen et al^33^ focused only on patients who received brachytherapy and adjusted for whether they also received EBRT (technique not specified). Chen et al^4^ included only patients with high-risk prostate cancer receiving EBRT (73%) or brachytherapy (14%) or both (13%), and Patel et al^32^ included only patients with lymph node–positive prostate cancer receiving EBRT. Patel et al^32^ was also the only study that described the dose of radiation therapy used and included only patients who received curative doses of 60 Gy (Table 1). Tchelebi et al^28^ included only patients who received definitive radiation therapy doses via EBRT (technique not specified) and/or brachytherapy; however, specific doses were not mentioned.
Bladder cancer	All 3 studies did not describe the radiation therapy technique used, for example, IMRT or 3D-CRT. Bajaj et al^34^ specifically analyzed curative doses of 60-70 Gy; Fischer-Valuck et al^35^ analyzed all doses between 50.4 and 75 Gy and standard curative fractionated doses of 59.4-64.8 Gy; and D'Rummo et al^36^ analyzed all doses >30 Gy and curative doses of 55-60 Gy (Table 1). Tchelebi et al^28^ included only patients who received definitive radiation therapy doses via EBRT (technique not specified) and/or brachytherapy; however, specific doses were not mentioned.
Other cancer types (<3 studies)	For all cancer groups studied by Tchelebi et al,^28^ only patients who received definitive radiation therapy doses via EBRT (technique not specified) and/or brachytherapy were included; however, specific doses were not mentioned (Table 2). Wang et al^37^ included patients with non-small cell lung cancer treated with nonconformal radiation therapy, 3D-CRT, and IMRT at doses between 59.4 and 74 Gy. Holliday et al^40^ included patients with hepatocellular carcinoma treated with brachytherapy or EBRT (technique not specified) with no mention of radiation therapy doses used. Haque et al^39^ included patients with glioblastoma treated with EBRT (technique not specified) treated with doses between 59.4 and 60 Gy. Amini et al^38^ included patients with anal squamous cell carcinoma treated with either IMRT or 3D-CRT but with no mention of doses used.

## Results

A total of 4356 studies were screened and assessed for eligibility, of which 20 studies (all retrospective cohort studies) were selected for inclusion (Fig. 1). Eighteen studies were from the United States, 2 were from Taiwan, and 1 was from South Korea. Of these studies, 7 looked at head and neck cancers (HNCs). The remaining studies covered cervical (4), prostate (4), bladder (3), lung (2), anal (2), brain (2), esophageal (1), hepatocellular (1), and pancreatic cancer (1). Only 1 study analyzed more than 1 cancer type, such as HNC, cervical, prostate, lung, anal, esophageal, brain, and pancreatic cancer.

### Meta-analyses

Eighteen studies reported HRs for overall survival. Sixteen of those studies grouped volume into quartiles or tertiles, and the remaining 2 studies dichotomized volume groups into either high- or low-volume radiation therapy facilities.

**Fig. 2:**
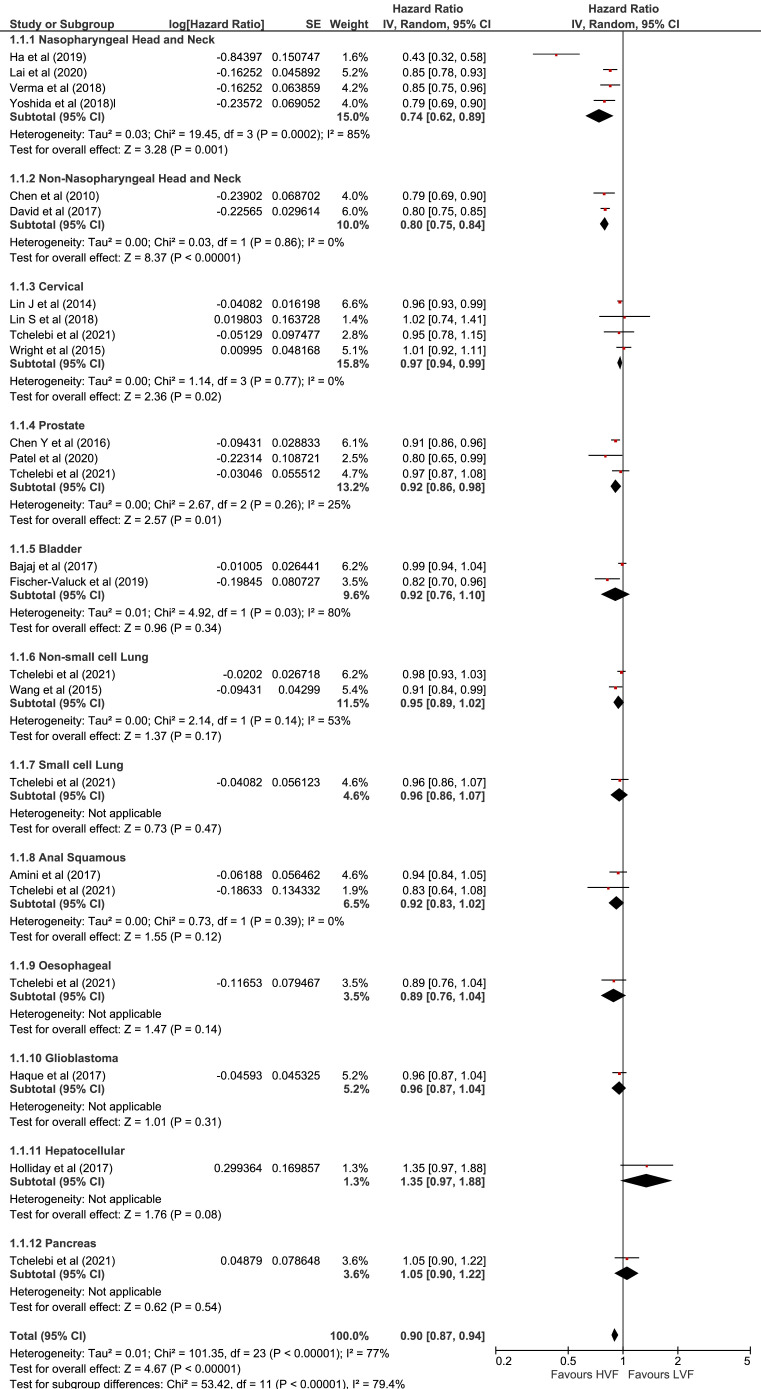
Forest plot. Pooled hazard ratios for overall survival comparing high-volume with low-volume radiation therapy facilities for 12 cancer subtypes. A total of 18 studies were included in the meta-analysis; Tchelebi et al^28^ appears under multiple cancer groups but represents only 1 study.

Overall pooled HR analyses of the 18 studies included in the meta-analysis in Fig. 2 show that receiving treatment at a high-volume radiation therapy facility (HVRF) was associated with a 10% lower chance of death compared with being treated at a low-volume radiation therapy facility (LVRF) (HR, 0.90; 95% CI, 0.87-0.94).

### Head and neck cancers (7 studies)

#### Results of outcomes

Seven studies were conducted on HNCs: 4 studies were on nasopharyngeal cancer^3^^,^^23^, ^24^, ^25^ and the remaining 3 were on non-nasopharyngeal HNC subsites (1 study on laryngeal cancer^26^; 1 on a combination of oropharyngeal, hypopharyngeal, laryngeal, and oral cavity cancers^27^; and 1 on multiple sites including nasopharyngeal cancer^28^).

Of the 7 HNC studies, 6 were included in the meta-analysis, which demonstrated a positive association between radiation therapy volume and survival outcomes. Of the 6 studies, all 4 nasopharyngeal cancer studies^3^^,^^23^, ^24^, ^25^ were included in the meta-analysis and had a pooled HR of 0.74 (95% CI, 0.62-0.89). Similarly, both non-nasopharyngeal HNC studies^26^^,^^27^ were included in the meta-analysis and had a pooled HR of 0.80 (95% CI, 0.75-0.84) (Fig. 2). The study by Tchelebi et al was not included in the meta-analysis because it did not stratify HNCs into either nasopharyngeal or non-nasopharyngeal subsites but rather combined both groups.^28^

Among the studies on nasopharyngeal cancers, Ha et al^23^ found the largest difference in outcomes between HVRFs (≥10 cases per year) and LVRFs (<10 cases per year), where 15.7% more patients (HR, 0.43; 95% CI, 0.32-0.57) survived at HVRFs compared with LVRFs over 120 months. The study also found that locoregional progression-free survival was 20.4% higher (HR, 0.34; 95% CI, 0.23-0.49) at HVRFs compared with LVRFs over 120 months. Lai et al^24^ found that survival improved with increasing volume quartiles (Q1, 1-85 cases; Q2, 86-274 cases; Q3, 275-651 cases; Q4, ≥652 cases over 7 years) (Table 1). The highest-volume quartile found up to a 47% higher chance of survival (HR, 0.63; 95% CI, 0.54-0.74) compared with the lowest-volume quartile over 180 months.^24^ Verma et al^3^ demonstrated that 6% more patients treated with curative doses at HVRFs (≥11 cases over 10 years) survived compared with LVRFs over 120 months (HR, 0.85; 95% CI, 0.75-0.96). Lastly, Yoshida et al^25^ showed that 9.5% more patients with nasopharyngeal cancer treated with curative doses survived when treated at HVRFs (≥3.36 cases per year) compared with LVRFs (<3.36 cases per year) over 120 months (HR, 0.77; 95% CI, 0.65-0.90) (Table 1).

For non-nasopharyngeal HNC subsites, Chen et al^26^ showed that 13% more patients with laryngeal cancer survived (HR, 0.79; 95% CI, 0.69-0.90) when treated at HVRFs (≥7.1 cases per year) compared with LVRFs (<7.1 cases per year) (both teaching and research facilities) over 4 years. David et al^27^ studied patients with laryngeal, oropharyngeal, and hypopharyngeal cancers and showed that 6.1% more patients receiving all doses survived when treated at HVRFs compared with LVRFs over 120 months (HR, 0.798; 95% CI, 0.753-0.845). However, volume thresholds were not described in that study.

Finally, for the study by Tchelebi et al,^28^ which was not included in the meta-analysis, absolute differences in survival were not described. However, the study did find that for patients with both nasopharyngeal and non-nasopharyngeal HNCs, all higher-volume quartiles (Q2, >1.5-3; Q3, >3-5.6; and Q4, ≥5.6 cases per year) showed an association with improved survival chances compared with the lowest-volume quartile (Q1, ≤1.5 cases per year) over 5 years (Q2 vs Q1: HR, 0.86; 95% CI, 0.82-0.96; Q3 vs Q1: HR, 0.83; 95% CI, 0.76-0.90; Q4 vs Q1: HR, 0.82; 95% CI, 0.75-0.90) (Table 1).

### Cervical cancer (4 studies)

#### Results of outcomes

Four studies were conducted on cervical cancer.^28^, ^29^, ^30^, ^31^ All 4 studies were included in the meta-analysis, which demonstrated an overall positive association between radiation therapy volume and survival outcomes (HR, 0.97; 95% CI, 0.94-0.99) (Fig. 2).

The study by J. L. Lin et al^29^ found that patients with stage IIB to IIIB cervical cancer treated at the highest-volume quartile radiation therapy facilities (Q4: ≥9.4 cases per year) had a 12% (HR, 0.96/quartile increase; 95% CI, 0.93-0.99; *P* < .0005) higher chance of survival compared with the lowest-volume quartile hospitals (Q1: ≤ 2.3 cases per year) over 100 months (Table 1). S. M. Lin et al^30^ found that the 5-year overall survival for patients with stage IB to IVA cervical cancer treated at the lowest-volume tertile (T1: ≤2 cases per year) was 37%, compared with 63% for the highest-volume tertile (≥5 cases per year) (*P* < .001). However, survival analysis found no overall hazard difference between the volume groups.^30^ On stratification, the study found that the chance of survival was 26% (HR, 0.74; 95% CI, 0.62-0.88; *P* < .01) higher in HVRFs (≥5 cases per year) compared with LVRFs (≤2 cases per year) for patients with tumors larger than 4 cm and 22% higher (HR, 0.78; 0.67-0.90; *P* < .01) for those with International Federation of Gynecology and Obstetrics stages IIB to IVA cervical cancer over a period of 96 months.^30^ The remaining 2 studies, by Tchelebi et al^28^ and Wright et al,^31^ found no association between radiation therapy volume and survival outcomes (Table 1).

### Prostate cancer (4 studies)

#### Results of outcomes

Four studies were conducted on prostate cancer.^4^^,^^28^^,^^32^^,^^33^ Three of those^4^^,^^28^^,^^32^ were included in the meta-analysis, which demonstrated a positive association between radiation therapy volume and survival outcomes (pooled HR, 0.92; 95% CI, 0.86-0.98) (Fig. 2). The study by A. B. Chen et al^33^ did not meet the inclusion criteria for the meta-analysis as it defined facility volume as a continuous variable.

Of the 3 studies included in the meta-analysis, Y. W. Chen et al^4^ found that 2% more patients with high-risk prostate cancer (HR, 0.91; 95% CI, 0.86-0.96) survived when treated at HVRFs (≥43 cases per year) compared with LVRFs (<43 cases per year) over 7 years (Table 1). Patel et al^32^ found that 9.1% more patients with lymph node–positive prostate cancer (HR, 0.80; 95% CI, 0.67-0.99) survived when treated at HVRFs (≥67 cases per year) compared with LVRFs (<67 cases per year) over 10 years. Tchelebi et al^28^ only found evidence of a radiation therapy volume–outcome association between the highest-volume quartile (Q4: ≥13 cases per year) and the lowest-volume quartile (Q1: ≤3.9 cases per year) (HR, 0.82; 95% CI, 0.74-0.91) for patients with nonmetastatic prostate cancer (Table 1). The study by A. B. Chen et al,^33^ which was not included in the meta-analysis, found no association between radiation therapy volume and survival outcomes over a period of 10 years and additional outcomes such as cancer recurrence for patients receiving brachytherapy (Table 1). Chen et al did, however, find a 6% (OR, 0.94; 95% CI, 0.91-0.98; *P* < .001) lower risk of complications requiring an invasive procedure for every 100 additional patients treated with brachytherapy within 2 years of radiotherapy^33^ (Table 1).

### Bladder cancer (3 studies)

#### Results of outcomes

Three studies were conducted on muscle-invasive bladder cancer^34^, ^35^, ^36^: Bajaj et al^34^ and D'Rummo et al^36^ included patients with both node-positive and node-negative bladder cancer. The study by Fischer-Valuck et al^35^ specifically looked at patients with only node-negative cancer.

Two of the studies on bladder cancer—Bajaj et al^34^ and Fischer-Valuck et al^35^—were included in the meta-analysis and found no overall association between radiation therapy volume and survival outcomes (pooled HR, 0.92; 95% CI, 0.76-1.10) (Fig. 2). The study by D'Rummo et al^36^ was not included in the meta-analysis because the study did not present any HRs.

Of the 2 studies included in the meta-analysis, the study by Bajaj et al^34^ did not find an association between radiation therapy volume and outcomes for patients with node-negative and node-positive bladder cancer treated at HVRFs (top 25th volume percentile) compared with LVRFs (bottom 75th percentile). The study did not enumerate volume cutoffs used to define HVRFs and LVRFs. The study by Fischer-Valuck et al^35^ found that patients with node-negative bladder cancer who received curative doses treated at HVRFs (≥6 cases in total over 10 years) lived a median length of 5.4 months longer (HR, 0.83; 95% CI, 0.73-0.96) when followed up over 120 months. Lastly, the study by D'Rummo et al,^36^ which was not included in the meta-analysis, found that 4.1% more patients survived when treated at HVRFs (≥14 cases cumulatively over 12 years) over 120 months (*P* = .013). When stratified by those who received curative doses, the volume–outcome relationship remained (*P* = .0081)^36^ (Table 1).

### Other cancer types (less than 3 studies per cancer group)

Studies in this category were organized into 6 groups: (1) lung cancer (2 studies^28^^,^^37^); (2) cancer of the anus (2 studies^28^^,^^38^); (3) brain cancer (2 studies^28^^,^^39^); (4) esophageal cancer (1 study^28^); (5) liver cancer (1 study^40^); and (6) pancreatic cancer (1 study^28^).

### Lung cancer (2 studies)

#### Results of outcomes

Two studies were conducted on lung cancer^28^^,^^37^: Wang et al^37^ studied non-small cell lung cancer (NSCLC) and Tchelebi et al^28^ studied both NSCLC and small cell lung cancer (SCLC) separately. Both studies were included in the meta-analysis and found no overall association between radiation therapy volume and survival outcomes for both NSCLC and SCLC (HRs, 0.95 [95% CI, 0.89-1.02] and 0.96 [95% CI, 0.86-1.07], respectively) (Fig. 2).

For patients with NSCLC, Wang et al^37^ found that 2.4% more patients treated at HVRFs (≥12 cases per year) survived compared with patients treated at LVRFs (<12 cases per year) (HR, 0.91; 95% CI, 0.84-0.99). Tchelebi et al^28^ only found improved survival for those treated at higher-volume quartiles—Q3: 5.5 to <8.5 cases per year (HR, 0.95; 95% CI, 0.9-0.99) and Q4: ≥8.5 cases per year (HR, 0.89; 95% CI, 0.84-0.93)—compared with quartile 1 (<2.7 cases per year) (Table 2).

For patients with SCLC, the study by Tchelebi et al^28^ found no association between radiation therapy volume and survival outcomes when comparing higher-volume quartiles (Q2: 0.5 to <0.9; Q3: 0.9 to <1.4; and Q4: ≥1.4 cases per year) to the lowest-volume quartile (Q1: <0.5 cases per year) (Table 2).

### Cancer of the anus (2 studies)

#### Results of outcomes

Both studies on cancer of the anus^28^^,^^38^ were included in the meta-analysis, which found no overall association between radiation therapy volume and survival outcomes (pooled HR, 0.92; 95% CI, 0.83-1.02) (Fig. 2).

The study by Amini et al^38^ showed that patients with stage I to III anal squamous cell carcinoma treated at low-volume (tertile 1 [T1]), intermediate-volume (T2), and high-volume (T3) radiation therapy facilities had a 5-year overall survival of 70.1%, 71.4%, and 74.6%, respectively. However, evidence of an association between radiation therapy volume and improved survival was only seen between the highest-volume (T3) and lowest-volume (T1) categories (HR, 0.79; 95% CI, 0.69-0.91).^38^ Importantly, the study did not enumerate volume cutoffs used for each tertile (Table 2). The study by Tchelebi et al^28^ found no association between radiation therapy volume and survival outcomes when comparing higher-volume quartiles (Q2: 0.3 to <0.5; Q3: 0.5 to <0.8; and Q4: ≥0.8 cases per year) to the lowest-volume quartile (Q1: <0.3 cases per year) (Table 2)

### Brain cancer (2 studies)

#### Results of outcomes

Only 1 brain cancer study, by Haque et al,^39^ was included in the meta-analysis and found no association between radiation therapy volume and survival outcomes (pooled HR, 0.96; 95% CI, 0.87-1.04) (Fig. 2). The study by Tchelebi et al^28^ was not included in the meta-analysis because the study did not specify what types of brain cancer were included in the study and therefore represented too broad a category for analyses.

Haque et al^39^ found that that the median months of survival of the highest-volume quartile and lowest-volume quartile for patients with glioblastoma were 16.5 months and 14.1 months (*P* < .001), respectively. A radiation therapy volume and outcome association was only demonstrated between the highest-quartile group (Q4: ≥9.1 cases per year) and the lowest-quartile group (Q1: ≤3.9 cases per year) (HR, 0.912; 95% CI, 0.835-0.005).^39^ The study by Tchelebi et al,^28^ which was not included in the meta-analysis, found no association between radiation therapy volume and survival outcomes when comparing higher-volume quartiles (Q2: >0.3-0.7; Q3: >0.7-1.2; and Q4: >1.2 cases per year) with the lowest-volume quartile (Q1: ≤0.3 cases per year).

### Esophageal cancer (1 study)

#### Results of outcomes

One study on esophageal cancer, by Tchelebi et al,^28^ was included in the meta-analysis. The study found no association between radiation therapy volume and survival outcomes when comparing higher-volume quartiles (Q2: 0.3 to <0.5; Q3: 0.5 to <1; and Q4: ≥1 cases per year) to the lowest-volume quartile (Q1: <0.3 cases per year) (Table 2).

### Hepatocellular cancer (1 study)

#### Results of outcomes

One study on hepatocellular cancer, by Holliday et al,^40^ was included in the meta-analysis. The meta-analysis found no volume–outcome relationship for patients with node-negative hepatocellular cancer receiving radiation therapy at high-volume (>144 cases per year) compared with low-volume facilities (≤144 cases per year) over 120 months (pooled HR, 1.35; 95% CI, 0.97-1.88) (Table 2).

### Pancreatic cancer (1 study)

#### Results of outcomes

One study on pancreatic cancer, by Tchelebi et al,^28^ was included in the meta-analysis and found no association between radiation therapy volume and survival outcomes (pooled HR, 1.05; 95% CI, 0.90-1.22) (Fig. 2). However, the study did find a volume–outcome relationship between the highest-volume quartile (Q4: >1.4 cases per year) and lowest-volume quartile (Q1: ≤0.3 cases per year) (HR, 0.84; 95% CI, 0.71-0.98) (Table 2).

## Discussion

To our knowledge, this is the first systematic review to evaluate evidence for an association between radiation therapy procedure volume at the hospital level and outcomes for patients with cancer. Our meta-analysis demonstrates a general trend suggesting that HVRFs have better patient outcomes compared with LVRFs. In terms of tumour category, a positive volume association was only seen in HNCs, prostate and cervical cancer.

The studies focused on a wide breadth of cancer types and subsites, with 7 of the 20 studies focused on head and neck cancers and 17 (85%) conducted in the United States. All 7 HNC studies showed a relationship between higher radiation therapy procedure volume and better survival outcomes at a hospital level. Ha et al^23^ additionally showed improvements in 5-year locoregional progression-free survival and lower rates of toxic effects (hematologic, mucositis, and xerostomia) at higher-volume facilities (Table 1). The meta-analysis showed that on conservation analysis, 2 of the 4 studies on prostate cancer,^4^^,^^32^ 1 of the 4 studies on cervical cancer,^29^ 1 of the 3 studies on muscle-invasive bladder cancer,^35^ and 1 of the 2 studies on non-small cell lung cancer^37^ demonstrated an association between higher-volume radiation therapy facilities and improved survival outcomes.

The reasons for the observed improvements in outcome at HVRFs are likely to be multifactorial. HVRFs may have better experience dealing with complex cases, which can contribute to improvements in treatment compliance and the prevention and management of cancer-related complications.^25^^,^^34^, ^35^, ^36^^,^^39^^,^^40^ In addition, a larger radiation therapy workforce capacity at these centers may help support peer review, as well as the ability to integrate new techniques in cancer management, which more rapidly translate into improved outcomes.^25^^,^^34^, ^35^, ^36^^,^^39^, ^40^, ^41^

High-volume radiation therapy facilities also ensure that greater expertise is gained in more complex techniques with steeper learning curves, such as brachytherapy.^42^^,^^43^ It has been demonstrated that contouring of target volumes, such as the gross tumor and nodal volumes, as well as organs at risk is prone to significant error and can have a detrimental effect on outcome.^44^ Therefore, reduction in variation and increased consistency of outlining through greater expertise at HVRFs may lead to better long-term outcomes.

However, increasing treatment volume through regionalization or specialization of radiation therapy is not guaranteed to deliver improvements in outcomes as observed for cancer surgery.^45^ Prolonged periods of treatment required for radiation therapy (up to 8 weeks) may not be feasible for all, and evidence suggests that the farther patients live from a radiation facility, the less likely they are to receive standard-of-care radiation at all.^46^, ^47^, ^48^, ^49^

From a policy perspective, we need to consider the trade-off between improvements in outcomes that may result from centralizing radiation therapy services and the effect this can have on access and equity in utilization of services. One consideration is for there to be a directive on minimum procedure volumes developed by the radiation therapy community, considering this evidence for particular types of cancer. Rather than restructuring to achieve “high” volumes, this would serve to restructure very low-volume centers and integrate care with more specialized high-volume centers, which has been advocated for surgery.^50^^,^^51^ To understand the implications of these closures on travel burden, equity, and outcomes, preimplementation modeling can be performed to ensure mitigation strategies can be considered.^52^

Where centralization is not feasible or likely to be acceptable, the data presented in this study provide support to address quality in lower-volume radiation therapy settings. To this end, efforts to integrate stand-alone facilities for quality assurance should be prioritized. Most radiation therapy is delivered via external beam radiation, where digital treatment plans can be developed, reviewed, and quality-assured at a distance. Although physician peer review is a requirement by accrediting organizations, most exempt single-physician practices without access to peers.^53^^,^^54^ Although yet to be standardized, other solutions include autosegmentation of target volumes as well as automated planning to reduce variation in plan quality through artificial intelligence algorithms. Meanwhile, during the COVID-19 pandemic, radiation oncology saw an increase in use of and access to telemedicine services,^55^, ^56^, ^57^ which can be leveraged for difficult cases, second opinions, and symptom management.^58^

There are several limitations to this review, which need to be considered when using it to support clinical or policy change. First, different volume definitions and categorizations were used between the studies. The result is that different studies within cancer types have different treatment-volume thresholds, making it difficult to determine what the optimum volume threshold should be to improve outcomes. Additionally, some studies demonstrated that certain centers defined as high-volume were undertaking very few procedures per annum (eg, <5 radiation therapy–managed patients per year) for tumors such as HNC subsites,^26^^,^^28^ cervical,^28^ bladder,^35^ lung,^28^ anal,^28^ esophageal,^28^ brain,^28^ and pancreatic^28^ cancer. If the threshold to translate into improved outcomes through the attainment of necessary competencies for complex tumors is more than 10 cases per year, then that volume effect will be missed.

Second, the generalizability of findings in this review is limited because most of the studies were conducted in the United States, with only 3 studies conducted in Asia (South Korea^23^ and Taiwan^24^^,^^30^). This is important because the relationship between hospital volume and outcome are intimately linked with the organization of health systems, availability of resources, varying disease burden, and socioeconomic realities in different countries.^18^

Third, the studies used a very limited set of outcome measures, with the majority assessing the relationship between radiation therapy volume and overall survival or mortality, which are crude measures of radiation therapy treatment quality and can be strongly influenced by confounders such as the general quality of the oncology care pathway and socioeconomic determinants. Only 2 of the 20 studies^23^^,^^33^ reported on additional outcomes such as locoregional progression-free survival, complications, and recurrence and more studies including these outcomes will need to be done to quantify a volume–outcome relationship. Additionally, other outcomes such as quality of life, function, or functional disability were not evaluated owing to the lack of availability of these outcome measures in routine data sets.^22^

Fourth, there may be several unmeasured confounders that could influence differences in outcomes between facilities of different volumes. For example, survival could be reflective of better downstream care of relapse or metastatic disease. Additionally, HVRFs are more likely to be comprehensive cancer centers, which have surgical and medical oncology expertise on site and offer more opportunities for research and trial participation; this has been shown to improve outcomes.^59^^,^^60^

Lastly, this review focused on the relationship between radiation therapy volume and patient outcomes at the facility level to examine health organization and structural factors. However, further studies assessing the relationship of radiation therapy volume and outcomes at an individual oncologist level may provide further insight into this discussion.

## Conclusion

To summarize, this meta-analysis shows that an association between radiation therapy procedure volume and outcome exists for most cancer types, with the strongest evidence in head and neck cancer. The study demonstrated a high prevalence of very-low-volume practice; however, heterogeneity in cohort selection and volume definitions prevents us from determining an ideal volume threshold to affect radiation therapy organization and policy development. Further studies are required in a broad range of health systems to understand better the trade-off between improving access to radiation therapy and supporting quality improvement. In this regard, consideration should be given to consolidate or augment support for very-low-volume practice.
